# 2-(2-Fluoro-4-nitro­phen­oxy)-3-nitro­pyridine

**DOI:** 10.1107/S1600536813034181

**Published:** 2013-12-24

**Authors:** Lili Cui, Xingquan He

**Affiliations:** a Department of Chemistry and Chemical Engineering, Changchun University of Science and Technology, Changchun 130022, People’s Republic of China

## Abstract

In the title compound, C_11_H_6_FN_3_O_5_, the dihedral angle between the aromatic rings is 72.4 (3)°. The NO_2_ groups form dihedral angles of 40.8 (2) and 4.8 (2)°, respectively, with the attached pyridine and benzene rings. The crystal structure features π–π stacking between centrosymmetrically related pairs of pyridine rings [centroid–centroid separation = 3.800 (3) Å].

## Related literature   

For applications and the biological activity of 2-phen­oxy­pyridine, see: Chao *et al.* (2013 ▶).
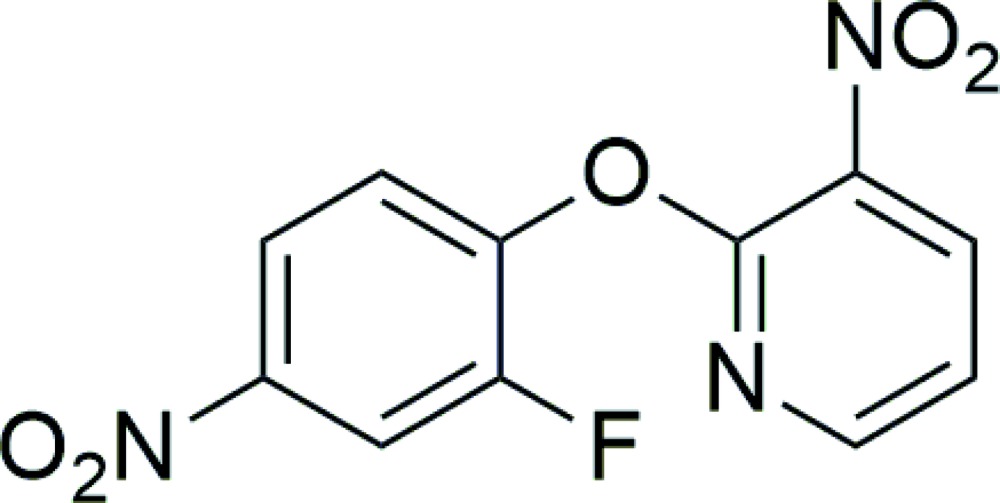


## Experimental   

### 

#### Crystal data  

C_11_H_6_FN_3_O_5_
*M* *_r_* = 279.19
Monoclinic, 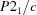

*a* = 7.5275 (15) Å
*b* = 21.804 (4) Å
*c* = 7.1681 (14) Å
β = 101.07 (3)°
*V* = 1154.6 (4) Å^3^
*Z* = 4
Mo *K*α radiation
μ = 0.14 mm^−1^
*T* = 293 K
0.41 × 0.36 × 0.22 mm

#### Data collection  

Rigaku R-AXIS RAPID IP diffractometer
Absorption correction: multi-scan (*ABSCOR*; Higashi, 1995 ▶) *T*_min_ = 0.945, *T*_max_ = 0.970
10606 measured reflections
2611 independent reflections
1351 reflections with *I* > 2σ(*I*)
*R*_int_ = 0.083

#### Refinement  

*R*[*F* ^2^ > 2σ(*F* ^2^)] = 0.062
*wR*(*F* ^2^) = 0.140
*S* = 0.94
2611 reflections
181 parameters
H-atom parameters constrained
Δρ_max_ = 0.21 e Å^−3^
Δρ_min_ = −0.26 e Å^−3^

### 

Data collection: *RAPID-AUTO* (Rigaku, 1998 ▶); cell refinement: *RAPID-AUTO*; data reduction: *RAPID-AUTO*; program(s) used to solve structure: *SHELXS97* (Sheldrick, 2008 ▶); program(s) used to refine structure: *SHELXL97* (Sheldrick, 2008 ▶); molecular graphics: *SHELXTL* (Sheldrick, 2008 ▶); software used to prepare material for publication: *SHELXTL*.

## Supplementary Material

Crystal structure: contains datablock(s) global, I. DOI: 10.1107/S1600536813034181/pk2504sup1.cif

Structure factors: contains datablock(s) I. DOI: 10.1107/S1600536813034181/pk2504Isup2.hkl

Supporting information file. DOI: 10.1107/S1600536813034181/pk2504Isup3.cml

Additional supporting information:  crystallographic information; 3D view; checkCIF report

## Figures and Tables

**Fig. 1.**
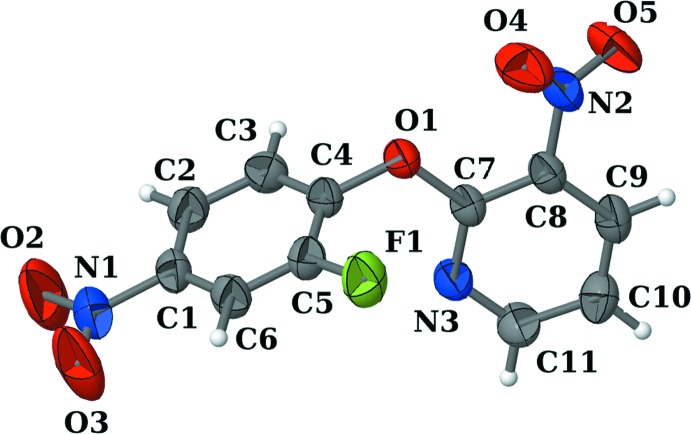
The molecular structure of the title compound. Displacement ellipsoids are shown at the 50% probability level.

## supplementary crystallographic information

### 1. Comment

2-Phenoxypyridines have been shown to be small molecule P2Y1 antagonists (Chao *et al.* 2013). Here, the crystal structure of 2-(2-fluoro-4-nitrophenoxy)- 3-nitropyridine is reported.

### 2. Experimental

To a solution of 2-chloro-3-nitropyridine (1.0 g, 6.3 mmol) and 2-fluoro-4-nitrophenol (1.48 g, 9.45 mmol) in toluene (35 ml) was added *N*,*N*-diisopropylethylamine (3.3 ml, 18.9 mmol). The reaction mixture was stirred for 48 h under reflux. The reaction mixture was concentrated *in vacuo*, diluted with water, and extracted with EtOAc. The organic phase was washed with brine, dried over anhydrous MgSO_4_, and concentrated *in vacuo* to yield the crude product as a solid. Purification by recrystallization from methanol gave the desired product, 2-(2-fluoro-4-nitrophenoxy)-3- nitropyridine (yellow solid, 0.80 g, 46%, 97.1–98.4 *^o^*C). ^1^H NMR (DMSO-*d6*, 300 Hz), 8.70 (dd, J = 8.1, 1.8 Hz, 1H), 8.47 (dd, J = 4.8, 1.8 Hz, 1H), 8.41 (dd, J = 10.2, 2.7 Hz, 1H), 8.25–8.20 (m, 1H), 7.80–7.74 (m 1H), 7.54–7.50 (m, 1H); ES–MS: 279.8 [(*M* + H^+^)]. Crystals of the title compound for X-ray diffraction were obtained by slow evaporation of MeOH/CH_2_Cl_2_ solution.

### 3. Refinement

All hydrogen atoms were fixed at calculated positions and refined by using a riding model with C—H = 0.93 Å and *U*_iso_(H) = 1.2*U*_eq_(C).

### Crystal data

**Table:**

C_11_H_6_FN_3_O_5_	*F*(000) = 568
*M**_r_* = 279.19	*D*_x_ = 1.606 Mg m^−^^3^
Monoclinic, *P*2_1_/*c*	Melting point = 370.1–371.4 K
Hall symbol: -P 2ybc	Mo *K*α radiation, λ = 0.71073 Å
*a* = 7.5275 (15) Å	Cell parameters from 7455 reflections
*b* = 21.804 (4) Å	θ = 3.1–27.7°
*c* = 7.1681 (14) Å	µ = 0.14 mm^−^^1^
β = 101.07 (3)°	*T* = 293 K
*V* = 1154.6 (4) Å^3^	Block, colourless
*Z* = 4	0.41 × 0.36 × 0.22 mm

### Data collection

**Table:**

Rigaku R-AXIS RAPID IP diffractometer	2611 independent reflections
Radiation source: fine-focus sealed tube	1351 reflections with *I* > 2σ(*I*)
Graphite monochromator	*R*_int_ = 0.083
Detector resolution: 0.1 pixels mm^-1^	θ_max_ = 27.5°, θ_min_ = 3.0°
oscillation scans	*h* = −9→9
Absorption correction: multi-scan (*ABSCOR*; Higashi, 1995)	*k* = −28→28
*T*_min_ = 0.945, *T*_max_ = 0.970	*l* = −9→8
10606 measured reflections	

### Refinement

**Table:**

Refinement on *F*^2^	Primary atom site location: structure-invariant direct methods
Least-squares matrix: full	Secondary atom site location: difference Fourier map
*R*[*F*^2^ > 2σ(*F*^2^)] = 0.062	Hydrogen site location: inferred from neighbouring sites
*wR*(*F*^2^) = 0.140	H-atom parameters constrained
*S* = 0.94	*w* = 1/[σ^2^(*F*_o_^2^) + (0.0498*P*)^2^] where *P* = (*F*_o_^2^ + 2*F*_c_^2^)/3
2611 reflections	(Δ/σ)_max_ < 0.001
181 parameters	Δρ_max_ = 0.21 e Å^−^^3^
0 restraints	Δρ_min_ = −0.26 e Å^−^^3^

### Special details

**Table:**

Geometry. All e.s.d.'s (except the e.s.d. in the dihedral angle between two l.s. planes) are estimated using the full covariance matrix. The cell e.s.d.'s are taken into account individually in the estimation of e.s.d.'s in distances, angles and torsion angles; correlations between e.s.d.'s in cell parameters are only used when they are defined by crystal symmetry. An approximate (isotropic) treatment of cell e.s.d.'s is used for estimating e.s.d.'s involving l.s. planes.
Refinement. Refinement of *F*^2^ against ALL reflections. The weighted *R*-factor *wR* and goodness of fit *S* are based on *F*^2^, conventional *R*-factors *R* are based on *F*, with *F* set to zero for negative *F*^2^. The threshold expression of *F*^2^ > σ(*F*^2^) is used only for calculating *R*-factors(gt) *etc*. and is not relevant to the choice of reflections for refinement. *R*-factors based on *F*^2^ are statistically about twice as large as those based on *F*, and *R*- factors based on ALL data will be even larger.

### Fractional atomic coordinates and isotropic or equivalent isotropic displacement parameters (Å^2^)

**Table:**

	*x*	*y*	*z*	*U*_iso_*/*U*_eq_	
C5	0.7119 (3)	0.37835 (11)	0.5574 (3)	0.0470 (6)	
C4	0.8649 (3)	0.34302 (12)	0.6136 (3)	0.0453 (6)	
C1	0.5475 (3)	0.29000 (12)	0.4494 (3)	0.0492 (6)	
C3	0.8592 (3)	0.28155 (12)	0.5825 (3)	0.0535 (7)	
H3	0.9634	0.2580	0.6169	0.064*	
C6	0.5501 (3)	0.35242 (12)	0.4752 (3)	0.0516 (7)	
H6	0.4464	0.3761	0.4385	0.062*	
C2	0.6968 (3)	0.25381 (13)	0.4988 (4)	0.0554 (7)	
H2	0.6904	0.2117	0.4772	0.066*	
C7	1.0406 (3)	0.39397 (11)	0.8728 (3)	0.0430 (6)	
C8	1.1950 (3)	0.42593 (11)	0.9561 (3)	0.0446 (6)	
C9	1.2093 (3)	0.44800 (12)	1.1372 (3)	0.0524 (6)	
H9	1.3124	0.4691	1.1956	0.063*	
C10	1.0688 (4)	0.43849 (13)	1.2313 (3)	0.0598 (7)	
H10	1.0732	0.4535	1.3536	0.072*	
C11	0.9221 (4)	0.40613 (13)	1.1387 (3)	0.0580 (7)	
H11	0.8268	0.3995	1.2018	0.070*	
F1	0.7237 (2)	0.43888 (7)	0.5831 (2)	0.0720 (5)	
O1	1.0278 (2)	0.37145 (9)	0.6923 (2)	0.0550 (5)	
O4	1.3069 (3)	0.45113 (12)	0.6873 (3)	0.0846 (7)	
O5	1.4970 (2)	0.43310 (12)	0.9461 (4)	0.0937 (8)	
O2	0.3673 (3)	0.20648 (13)	0.3537 (5)	0.1251 (11)	
O3	0.2448 (3)	0.29424 (13)	0.3109 (4)	0.1255 (12)	
N3	0.9075 (3)	0.38343 (10)	0.9628 (3)	0.0508 (5)	
N2	1.3435 (3)	0.43708 (10)	0.8543 (4)	0.0593 (6)	
N1	0.3735 (3)	0.26136 (14)	0.3662 (4)	0.0716 (7)	

### Atomic displacement parameters (Å^2^)

**Table:**

	*U*^11^	*U*^22^	*U*^33^	*U*^12^	*U*^13^	*U*^23^
C5	0.0507 (13)	0.0405 (14)	0.0488 (12)	−0.0032 (11)	0.0074 (11)	−0.0045 (10)
C4	0.0391 (12)	0.0565 (16)	0.0385 (11)	−0.0039 (11)	0.0029 (10)	−0.0049 (10)
C1	0.0444 (13)	0.0523 (16)	0.0473 (13)	−0.0054 (11)	−0.0005 (11)	−0.0089 (11)
C3	0.0452 (13)	0.0559 (17)	0.0568 (14)	0.0103 (11)	0.0034 (12)	−0.0036 (12)
C6	0.0428 (13)	0.0531 (17)	0.0541 (14)	0.0053 (11)	−0.0028 (11)	−0.0027 (11)
C2	0.0579 (15)	0.0461 (16)	0.0600 (15)	0.0013 (12)	0.0060 (13)	−0.0083 (11)
C7	0.0374 (12)	0.0461 (14)	0.0429 (11)	−0.0031 (10)	0.0009 (10)	−0.0003 (10)
C8	0.0356 (11)	0.0426 (14)	0.0526 (13)	−0.0010 (10)	0.0008 (11)	0.0020 (10)
C9	0.0477 (13)	0.0484 (15)	0.0546 (14)	−0.0047 (11)	−0.0060 (12)	−0.0031 (11)
C10	0.0678 (17)	0.0625 (18)	0.0448 (13)	−0.0060 (14)	0.0003 (13)	−0.0058 (12)
C11	0.0575 (15)	0.0730 (19)	0.0447 (13)	−0.0122 (14)	0.0126 (12)	−0.0008 (12)
F1	0.0736 (10)	0.0425 (9)	0.0955 (11)	−0.0038 (7)	0.0051 (9)	−0.0074 (8)
O1	0.0392 (9)	0.0785 (13)	0.0472 (9)	−0.0119 (8)	0.0080 (7)	−0.0158 (8)
O4	0.0682 (13)	0.116 (2)	0.0747 (13)	−0.0145 (13)	0.0261 (11)	0.0135 (13)
O5	0.0355 (10)	0.121 (2)	0.1207 (18)	−0.0093 (11)	0.0043 (11)	−0.0072 (15)
O2	0.0874 (18)	0.0756 (19)	0.202 (3)	−0.0328 (14)	0.0027 (18)	−0.0372 (19)
O3	0.0584 (14)	0.109 (2)	0.183 (3)	−0.0043 (14)	−0.0421 (17)	−0.0136 (19)
N3	0.0449 (11)	0.0617 (14)	0.0457 (10)	−0.0105 (10)	0.0086 (9)	−0.0026 (9)
N2	0.0392 (11)	0.0564 (15)	0.0805 (16)	−0.0078 (10)	0.0073 (11)	−0.0048 (12)
N1	0.0568 (15)	0.0735 (19)	0.0797 (16)	−0.0166 (13)	0.0011 (13)	−0.0196 (14)

### Geometric parameters (Å, º)

**Table:**

C5—F1	1.333 (3)	C7—C8	1.388 (3)
C5—C6	1.369 (3)	C8—C9	1.369 (3)
C5—C4	1.380 (3)	C8—N2	1.468 (3)
C4—C3	1.358 (3)	C9—C10	1.375 (4)
C4—O1	1.393 (3)	C9—H9	0.9300
C1—C2	1.363 (3)	C10—C11	1.370 (3)
C1—C6	1.373 (3)	C10—H10	0.9300
C1—N1	1.470 (3)	C11—N3	1.339 (3)
C3—C2	1.392 (3)	C11—H11	0.9300
C3—H3	0.9300	O4—N2	1.215 (3)
C6—H6	0.9300	O5—N2	1.218 (2)
C2—H2	0.9300	O2—N1	1.200 (3)
C7—N3	1.312 (3)	O3—N1	1.209 (3)
C7—O1	1.370 (3)		
			
F1—C5—C6	119.9 (2)	C9—C8—C7	119.4 (2)
F1—C5—C4	118.9 (2)	C9—C8—N2	118.9 (2)
C6—C5—C4	121.3 (2)	C7—C8—N2	121.6 (2)
C3—C4—C5	120.3 (2)	C8—C9—C10	119.0 (2)
C3—C4—O1	120.3 (2)	C8—C9—H9	120.5
C5—C4—O1	119.3 (2)	C10—C9—H9	120.5
C2—C1—C6	123.3 (2)	C11—C10—C9	117.6 (2)
C2—C1—N1	119.0 (2)	C11—C10—H10	121.2
C6—C1—N1	117.7 (2)	C9—C10—H10	121.2
C4—C3—C2	119.8 (2)	N3—C11—C10	124.0 (3)
C4—C3—H3	120.1	N3—C11—H11	118.0
C2—C3—H3	120.1	C10—C11—H11	118.0
C5—C6—C1	117.1 (2)	C7—O1—C4	116.05 (18)
C5—C6—H6	121.4	C7—N3—C11	117.8 (2)
C1—C6—H6	121.4	O4—N2—O5	124.2 (2)
C1—C2—C3	118.2 (3)	O4—N2—C8	118.75 (19)
C1—C2—H2	120.9	O5—N2—C8	117.0 (2)
C3—C2—H2	120.9	O2—N1—O3	123.3 (3)
N3—C7—O1	118.66 (19)	O2—N1—C1	118.2 (3)
N3—C7—C8	122.1 (2)	O3—N1—C1	118.4 (3)
O1—C7—C8	119.2 (2)		
